# Prevalence of Child Maltreatment in Adults With Congenital Heart Disease and Its Relationship With Psychological Well-Being, Health Behavior, and Current Cardiac Function

**DOI:** 10.3389/fpsyt.2021.686169

**Published:** 2021-07-26

**Authors:** Phileas J. Proskynitopoulos, Ivo Heitland, Alexander Glahn, Johann Bauersachs, Mechthild Westhoff-Bleck, Kai G. Kahl

**Affiliations:** ^1^Department of Psychiatry, Social Psychiatry and Psychotherapy, Hannover Medical School, Hanover, Germany; ^2^Department of Cardiology and Angiology, Hannover Medical School, Adult Congenital Heart Centre, Hanover, Germany

**Keywords:** congenital heart disease, childhood maltreatment, quality of life, childhood trauma questionnaire, adults with congenital heart disease, ACHD, NYHA, CTQ

## Abstract

**Background:** The prevalence of child maltreatment in adults with congenital heart disease (ACHD) has not been assessed so far. Child maltreatment is a major risk factor for unfavorable behavioral, mental, and physical health outcomes and has been associated with decreased quality of life. Given the increased survival time of ACHD, it is essential to assess factors that may worsen the quality of life and interact with classical cardiovascular risk factors and mental well-being.

**Methods:** In a cross-sectional study, 196 ACHD (mean age 35.21 ± 11.24 y, 44,4% female, 55.6% male) completed a thorough psychiatric and cardiac evaluation. Child maltreatment was assessed using the Childhood Trauma Questionnaire (CTQ) and rates were compared to already existing data from the German general population. Further psychological measurements included the WHO Quality of Life Questionnaire, Hospital Anxiety and Depression Scale (HADS) and assessment of lifestyle factors (exercise, smoking, alcohol consumption, body mass index). To identify a relationship between current cardiac function and child maltreatment, we used logistic regression.

**Results:** ACHD reported significantly higher rates of emotional neglect and emotional abuse and sexual abuse and lower rates of physical neglect when compared to the general German population. In addition, total CTQ-scores, emotional abuse, emotional neglect, physical abuse, and sexual abuse correlated with symptoms of depression, anxiety, and negatively correlated with QoL. Furthermore, CTQ scores contributed significantly in predicting higher New York Heart Association (NYHA) scores (*p* = 0.009).

**Conclusion:** Child maltreatment is more common in ACHD and associated with decreased quality of life and depression and anxiety. Furthermore, we found evidence that self-reported child maltreatment is associated with decreased cardiac function. Given the longer survival time of patients with ACHD, identifying factors that may negatively influence the disease course is essential. The negative consequences of child maltreatment may be the subject of psychosocial interventions that have demonstrated efficacy in treating posttraumatic stress disorders.

## Introduction

Advancements in medical and surgical care lead to an increasing population of adults with congenital heart disease (ACHD). With an increase in life expectancy, CHD's long-term effects on both the quality of life (QoL) and the development of psychiatric disorders caused significant scientific interests. Even though the authors of a large-scale study regarded QoL in ACHD as “generally good” (1), it is crucial to consider physical, social, and psychological aspects in QoL assessment as mental disorders greatly influence a patient's mental health and well-being (2). For example, reduced QoL is present even in mild forms of major depressive disorder (3).

Although not uniformly, most studies have reported an increased risk and under-treatment of mood and anxiety disorders in ACHD (1, 3–6). Current mood and anxiety disorders have been associated with decreased quality of life in ACHD, and even mild forms of major depressive disorder led to reduced QoL in ACHD (7).

Parental mental health conditions are a well-known risk factor for child maltreatment that impacts families with children suffering from CHD. Studies have shown (8–14) that the child's burden concerning CHD is shared between family members, affecting the children's and parents' mental health and the child-parental interaction. In one study, CHD was identified as a substantial risk factor for mental health problems in children with CHD and their respective mothers (8). Depression, anxiety, somatization, and hopelessness were frequently found in parents of children with CHD, with mothers reporting more symptoms than fathers (9, 10). Multivariate analysis showed that distress, hopelessness, and the financial situation were more important in explaining diminished QoL than the children's heart condition (9). Another meta-analysis described a strong association between maternal depression and malicious maternal behavior in CHD (11). Furthermore, parental depression increases the risk of coercive and hostile parenting style as well as corporal punishment (12), while it was also shown that an improvement in maternal depression leads to an improvement in child depression and the child's functionality as assessed with the Columbia impairment scale (13). A study in women showed that child maltreatment was associated with poor overall health, physical, emotional, and functional disability, distressing physical symptoms, and a greater health risk behavior (14). In summary, having to care for a child with CHD is a significant burden for a family as it is associated with constant worrying about the child's health and future, frequent visits to health care institutions, frequent health care interventions and a potentially associated financial strain. These circumstances add to decreased parental capacities and increased likelihood for neglectful or malicious behavior.

The higher prevalence of mental health issues in CHD children and parents and its negative impact on child-parent interaction suggest that adults with CHD report more traumatic experiences and abusive behavior from their parents when compared to the general population. Therefore, the objectives of the present study are:

1. To assess the prevalence of self-reported child maltreatment in a cohort of 196 ACHD patients using the Childhood Trauma Questionnaire
2. To compare the frequency of child maltreatment in ACHD with the prevalence estimates from the general population in Germany
3. To assess if child maltreatment is associated with symptoms of depression and anxiety, and with quality of life
4. To assess if self-reported child maltreatment is associated with worse cardiac function.

We hypothesized that the prevalence of child maltreatment is higher in ACHD patients than in the general population and that child maltreatment is associated with decreased quality of life and increased depression and anxiety.

## Materials and Methods

### Participants

The PSYConHEART study is an ongoing project aiming at examining morbidity and mortality factors in ACHD (7). The study protocol conformed to the ethical guidelines of the declaration of Helsinki and was approved by the local ethics committee. All patients gave their informed written consent before study entry. Patients were recruited from the ACHD outpatient clinic of the Dep. of Cardiology and Angiology at the Hannover Medical School (Hanover, Germany). The inclusion criteria were as follows: (1) structural congenital heart disease, (2) ability to read and complete the informed consent form and questionnaires in German, and (3) age of 18 or older. Exclusion criteria were pregnancy and unstable cardiac conditions. Currently, 215 ACHD patients were recruited, of whom 19 had to be excluded due to incomplete data from of at least one of the questionnaires. The final sample consists of 109 male and 87 female patients. Details of the underlying heart disease and treatment are given in Table 1.

**Table 1:** Descriptive statistics regarding socio-demographics, questionnaires, and type of cardiac malformation of the current sample.

	**Study sample (*N* = 196)**
Males/Females (*N*/%)	109/87 (44.4%/55.6%)
Age (M/SD)	35.21 ± 11.24y
Smoker (*N*/%)	53 (27%)
Mean BMI	25.39 ± 4.97
Exercise level	3.28 ± 1.76
In a relationship	112 (57.14%)
**School years**
Up to 9	31 (15.8%)
Up to 11 (junior high school)	94 (48%)
Up to 13 (secondary school)	71 (36.2%)
HADS-A (M/SD)	4.67 ± 3.36
HADS-D (M/SD)	3.48 ± 3.63
**NYHA score (*****N*****/%)**
I	150 (76.5%)
II	35 (17.9%)
II	11 (5.6%)
IV	0
**Bethesda score (*****N*****/%)**
1	106 (54.1%)
2	53 (27.0%)
3	37 (18.9%)
**Cardiac malformation (*****N*****)**
Simple shunts	15
Atrioventricular septal defect	10
Mitral valve disease	4
Anomalous pulmonary venous connection	1
Bicuspid aortic valve	24
Subaortic stenosis	6
Coarctation	23
Congenital pulmonary stenosis	8
Double chambered right ventricle	2
Tetralogy of Fallot	30
Ebstein anomaly	7
Marfan Syndrome	16
D-Transposition: Atrial switch	24
D-Transposition: Arterial switch	1
Congenital corrected transposition	3
Fontan type circulation	16
Eisenmenger Syndrome	6

*BMI, body mass index; HADS-A, hospital anxiety and depression scale, anxiety subscale; HADS-D, hospital anxiety and depression scale, depression subscale; NYHA, New York heart association score; M, mean; SD, standard deviation. All numbers are rounded to two decimal digits*.

### Assessment of Child Maltreatment

Child maltreatment was assessed using the Childhood Trauma Questionnaire (15). The CTQ is a well-established instrument with a high internal consistency [Cronbach's alpha = 0.95, (16)]. This self-administered questionnaire covers childhood adversities in five areas: emotional abuse, physical abuse, sexual abuse, emotional neglect, and physical neglect.

With reference to Walker et al. the items used to construct the five maltreatment scales were derived from the following definitions of abuse and neglect (14). Sexual abuse was defined as “sexual contact or conduct between a child younger than 17 years of age and an adult or older person (at least 5 years older than the child).” Physical abuse was defined as “bodily assaults on a child by an adult or older person that posed a risk of, or resulted in, injury.” Emotional abuse was defined as “verbal assaults on a child's sense of worth or well-being or any humiliating or demeaning behavior directed toward a child by an adult or older person.” Physical neglect was defined as “the failure of caretakers to provide for a child's basic physical needs, including food, shelter, clothing, safety, and health care” (poor parental supervision was also included if it placed a child's safety in jeopardy). Emotional neglect was defined as “the failure of caretakers to meet the children's basic emotional and psychological needs, including love, belonging, nurturance, and support.” Groups (with either form of maltreatment/ no maltreatment) were constructed similar to the thresholds given in Walker et al. (14). To compare our sample with the German population, we used the data described by Iffland et al. (17). In their paper, the authors also used the thresholds presented by Walker et al. (14).

### Assessment of Health Behavior and Symptoms of Anxiety and Depression and Quality of Life

Symptoms of anxiety and depression were assessed using the Hospital Anxiety and Depression Scale [HADS (18)] and the World Health Organization Quality of Life [WHO-QoL-BREF (19)] questionnaire for the assessment of quality of life. All three questionnaires have a high internal consistency [Cronbach's alpha: HADS-A = 0.83; HADS-D = 0.82 (20); WHO-QoL-BREF =0.87 (21)]. Participants completed a demographic survey that included educational, marital and employment status, smoking habits (expressed as pack-years) and alcohol drinking behavior (expressed as drinks consumed per week). Physical activity and exercise were assessed using a 6-point Likert scale as described elsewhere (22). Alcohol consumption was assessed as drinks per week and cigarette smoking by pack-years (cigarettes per day x years smoking)/20).

### Assessment of Cardiac Disease

Echocardiography was performed in all patients to evaluate cardiac morphology and function by an experienced cardiologist. Cardiac defects were categorized regarding their complexity as simple, moderate, or great using the Warnes classification Bethesda score (23). Functional status was assessed by a cardiologist and determined according to the New York Heart Association (NYHA) class (24).

### Statistical Analysis

Data were analyzed using SPSS (Version 26, IBM, Armonk, NY, 2020). Group comparisons concerning the prevalence of childhood traumata (ACHD vs. general population) were determined by performing the Chi-square tests. We categorized values of the CTQ and its subscores using the threshold/cut-off described by Walker et al. (14), which was also used in a representative sample of the general German population presented by Iffland et al. (17).

Pearson's correlation analysis was performed to demonstrate associations between total child maltreatment as assessed via total CTQ scores, different trauma types as assessed via CTQ subscores, behavioral (drinking and smoking behavior as well as physical exercise and BMI) and mental health factors (anxiety, depression and quality of life).

Logistic regression analysis was performed to assess the relationship between child maltreatment as measured by the overall CTQ score and current cardiac symptoms as determined via the NYHA score. To that end, we compared asymptomatic patients (i.e., NYHA I) with symptomatic patients (i.e., NYHA II and III). All data are shown as mean ±SD. An alpha of 0.05 was used for all analyses.

## Results

One hundred and ninety-six patients completed the questionnaires and fulfilled our inclusion criteria (109 males and 87 females/44.4% female, average age 35.21 years ± 11.24. According to the NYHA score, 150 patients were asymptomatic (NYHA I), and 46 were symptomatic (35 NYHA II and 11 NYHA III, no NYHA IV patients). For the demographics and details regarding underlying heart diseases, see Table 1.

### Prevalence of Child Maltreatment in ACHD

Compared to a sample from the general German population using the same thresholds, patients with ACHD had significantly higher rates of emotional abuse [*χ*^2^_(1)_ = 45.57 *p* < 0.001] and emotional neglect [*χ*^2^_(1)_ = 60.11, *p* < 0.001]. Also, sexual abuse was more prevalent in the ACHD cohort [*χ*^2^_(1)_ = 7.32, *p* = 007] while physical neglect was more prevalent in the German population [*χ*^2^_(1)_ = 39.98, *p* < 0.001]. There was no difference regarding physical abuse [*χ*^2^_(1)_ = 0.30, *p* = 0.58] (see Table 2).

**Table 2:** Prevalence of child maltreatment in adults with congenital heart disease: comparison to data from the German population (25).

	**ACHD**	**German population**			
	**X/N**	**%**	**X/N**	**%**	***p***
CTQ-emotional abuse	51/196	26.0	254/2500	10.2	<0.001
CTQ-emotional neglect	68/196	34.6	348/2500	13.9	<0.001
CTQ-physical abuse	21/196	10.7	301/2500	12	n.s.
CTQ-physical neglect	49/196	25	1210/2500	48.8	<0.001
CTQ-sexual abuse	22/196	11.2	156/2500	6.2	0.007

*ACHD, adults with congenital heart disease; CTQ, childhood trauma questionnaire; X, respective number of participants; N, total number of participants*.

### Correlation of Emotional Neglect, Emotional Abuse, and Sexual Abuse on Quality of Life, Psychological Well-Being, and Health Behavior

Using Pearson's correlation analysis (Table 3), overall CTQ-scores, emotional abuse, emotional neglect, physical abuse, and sexual abuse were correlated with anxiety symptoms and negatively correlated with QoL. Furthermore, quality of life was negatively and depression was positively correlated with physical neglect.

**Table 3:** Results of correlation analysis (Pearson's r) between child maltreatment, psychological well-being and health behavior (*N* = 196).

	**HADS-D**	**HADS-A**	**QoL**	**BMI**	**Exercise**	**Drinks per week**	**Smoking behavior**
CTQ-total score	0.32^**^	0.33^**^	−0.30^**^	0.14	−0.06	−0.18^**^	0.10
CTQ-emotional abuse	0.28^**^	0.27^**^	−0.26^**^	0.16^*^	−0.11	−0.14	0.10
CTQ-emotional neglect	0.32^**^	0.27^**^	−0.32^**^	0.10	−0.01	−0.14^*^	0.11
CTQ-physical abuse	0.22^**^	0.24^**^	−0.15^*^	0.14	−0.04	−0.12	0.08
CTQ-physical neglect	0.15^*^	0.14	−0.19^**^	0.13	−0.02	−0.14^*^	0.13
CTQ-sexual abuse	0.26^**^	0.29^**^	−0.20^**^	0.00	−0.08	−0.11	−0.03

*BMI, body mass index; CTQ, childhood trauma questionnaire; HADS-A, hospital anxiety and depression scale, anxiety subscale; HADS-D, hospital and anxiety and depression, depression subscale; N, number of participants; QoL, quality of life. All numbers are rounded to two decimal digits*.

** *p < 0.01*.

* *p < 0.05*.

Total CTQ and the emotional neglect and physical neglect subscore were negatively associated with drinks per week. Emotional abuse was negatively associated with BMI.

### Results of the Regression Analysis

To assess the association between child maltreatment and current cardiac symptomatology, we used a logistic regression model with CTQ total score as the independent variable and NYHA class (asymptomatic [NYHA I] vs. symptomatic [NYHA II and III collapsed]) as the dependent variable. The model was statistically significant [*χ*^2^_(1)_ = 6.85, *p* = 0.009]. Goodness-of-fit was assessed using the Hosmer-Lemeshow-Test, indicating a good model fit [*χ*^2^_(8)_ = 5.88, *p* = 0.661]. CTQ contributed significantly in predicting NYHA severity (*p* = 0.009). Higher total CTQ scores increased the risk of having a higher NYHA class (OR = 1.031, 95%-CI [1.008, 1.055]).

## Discussion

In the current study, adults with CHD more frequently reported emotional neglect, emotional abuse, and sexual abuse compared to a representative sample from the German population. Furthermore, there was a significant positive association between overall child maltreatment, emotional abuse, emotional neglect, physical abuse, and sexual abuse on the one hand and decreased quality of life, increased anxiety and increased depression on the other hand. Moreover, there was a negative association between physical neglect and quality of life. Using logistic regression, we identified a negative relationship between child maltreatment and current cardiac function as assessed via the NYHA score.

However, and contrary to our hypothesis, physical neglect was significantly more frequent in the general German population, which could have numerous reasons. As Iffland et al. (17) point out, a different study on the psychometric properties of the German version of the CTQ argued that the physical neglect subscale has weak psychometric properties, is highly correlated with the other subscales, and has a weak internal consistency (17, 26). Furthermore, a study from 2018 has shown that older respondents who had experienced privation during the (post-) war years were more likely to report physical neglect (27). This could partly explain the difference between our sample and the findings reported by Iffland et al. (17), since their study was conducted in 2013 and the average age of all participants was 50.66 (SD = 18.56) as opposed to 35.21 years ± 11.24 in the present study. Nonetheless, comparisons of physical neglect should, therefore, only be made with caution.

Since we identified significantly higher emotional neglect, abuse and sexual abuse in ACHD, this could suggest that ACHD suffer from poly-victimization or multiple victimizations as defined by Finkelhor et al. (28). Several studies have shown that victims of poly-victimization have a higher risk for trauma symptoms (28), posttraumatic stress disorder, and depression (29, 30). One study (25) has described a dose-response relation between the number of potentially traumatic events experienced and psychological distress. Participants who experienced an average of 10 types of trauma during their lives were at the most significant risk for PTSD scores and engagement in risky behavior such as substance use and suicidal behaviors. One meta-analysis and systematic review comprising 124 articles has concluded that the current evidence suggests a causal relationship between child maltreatment (non-sexual) and a range of mental disorders, substance abuse, suicidal behavior, and risky sexual behavior (31). Furthermore, a study on students in grades 9–12 has shown that past-year sexual violence victimization was related to health risk behaviors such as substance abuse, injury, risky sexual behavior, suicidal ideation, and feelings of sadness and hopelessness (32). It is well-established that depression is a risk factor for non-compliance with medical treatment: in one rigorous study (33), the odds were three times higher that patients suffering from depression will be non-compliant when compared with healthy controls. Furthermore, one randomized controlled trial study in recently hospitalized cardiac patients has shown that improvement of depression was consistently and independently associated with superior self-reported adherence to medications and secondary prevention behaviors across a 6-month span. Of note, in this study, improvement in anxiety did not show the same effect (34). Thus, it is very important to identify especially those who were the victim of multiple traumatization as they are at a high risk of developing depression. This could then influence therapy adherence and, subsequently, long-term prognosis.

The effect mentioned above of child maltreatment on therapy adherence could even translate into worse cardiac function and thereby increased mortality. In our study, using logistic regression, overall CTQ scores were associated with higher NYHA class. In ACHD, NYHA class predicts mid- to long-term mortality (35). Our results, therefore, suggest that child maltreatment could negatively influence morbidity and mortality in this specific patient population. One possible mechanism for this association could be the influence of traumatization and depression on therapy adherence. Therefore, it is reasonable to assume that therapy adherence could negatively influence long-term outcome and NYHA class. However, to the best of our knowledge, no study has yet identified the relationship between therapy adherence, mental disorders and cardiac function in ACHD. Rigorous studies investigating this proclaimed relationship are therefore needed.

### Parental Factors

This study found a strong association between child maltreatment, emotional abuse, emotional neglect, physical abuse, and sexual abuse with higher anxiety and depression and lower QoL. Emotional neglect is considered to have a high prevalence rate and a significant impact on the child's health and development. Emotional neglect is determined by different factors such as child stigmata (e.g., a chronic disease), the well-being of parents, social factors (e.g., family income) and even culture and society factors (36). Most of the factors that influence the childhood neglect risk are parent-related (36–38). As disability can be regarded as one child-related risk factor for child neglect (39), one can assume that parents-related factors are more aggravating. This, in turn, could explain the higher prevalence of emotional neglect found in our study.

Indeed, some studies have shown that major depressive disorder poses a risk for child abuse (40) while some symptoms frequently occurring in anxiety and major depressive disorder are also associated with child neglect (37, 38, 41). Hence, the risk for mental disorders in both parents and the child caused by the child's CHD could partly explain the higher prevalence of emotional abuse and neglect reported in our study. Nonetheless, our report is limited because we did not test the patients' parents for mental disorders. In future studies, it would therefore be essential to assess mental disorders and childhood abuse in both children and their parents to assess the CHD's risk on the patients' mental health and potential abuse and neglect.

Since we have also identified a higher rate of experienced sexual abuse in patients with ACHD, it is plausible to raise the question if CHD poses a risk for sexual abuse. One could assume that childhood trauma and especially sexual abuse would increase the risk of developing a chronic illness later in adulthood, which was recently shown in an extensive surveillance system study analyzing 86,968 respondents from a population of 32 million people. In this study, the authors were able to show that young adults with high scores in adverse childhood experiences were at higher risk for the development of early-onset disease (42). Interestingly, parental factors and especially a problematic mother-daughter relationship put children at a greater risk for sexual abuse (43). As we have discussed above, some studies have shown that maternal major depressive disorder and specific psychiatric symptoms classified in the mood and anxiety spectrum are associated with a higher risk for abuse of the respective child (37, 38, 40, 41). Sharing this common factor, namely a possible hazardous and problematic mother-child relationship, the increased prevalence identified in our study may be partly associated with this relationship's structure. However, we cannot conclude a possible causal relationship. It is still important to highlight the importance of further studies that could examine the risk of early-onset chronic illnesses and an association with childhood sexual abuse.

One conclusion that can be drawn from the results is that parents of children with CHD may be better integrated into the treatment process and should receive social and psychological help when necessary. In addition, helping parents cope with their children's disease may lead to decreased rates of emotional neglect. Thus, emotional neglect (and abuse) would be a modifiable variable that could reduce mental health burden in ACHD when addressed through early interventions of the patients' parents.

Some factors investigated in our study that are associated with child maltreatment influence morbidity and mortality, namely higher BMI, increased alcohol consumption and depression. In our study, BMI was negatively associated with emotional abuse. Contrary to the public belief that higher BMI is generally associated with higher mortality, in ACHD, higher body mass index is generally regarded as being associated with lower mortality (44, 45), especially in symptomatic patients with complex underlying cardiac defects, suggesting that cardiac cachexia could influence mortality. In a population-based study from the UK, BMI had a j-curved association to mortality in cardiovascular disease, among others, i.e., the mortality was higher in below and above average BMI values (46). However, it is essential to highlight that BMI does not directly translate into obesity and that lower BMI is associated with higher mortality, especially in patients suffering from CHD. Thus, the associations between BMI and emotional neglect and abuse in this patient collective need to be further elucidated and differentiated for cachexia, physiological BMI, and obesity.

Increased alcohol consumption is associated with higher mortality (47). In ACHD, one study has shown that self-reported depressive symptoms are associated with increased alcohol and tobacco use (48). In our study, however, we have found that overall CTQ scores and emotional neglect are negatively associated with drinks per week, i.e., the higher the reported child maltreatment the lower the number of drinks per week. Our study extends the existing literature and demonstrates that child maltreatment and its relationship to alcohol consumptions need further investigations. Nonetheless, a recent meta-analysis by Hughes et al. (49) demonstrated that child maltreatment is a major risk factor for many health conditions, such as physical inactivity, overweight, diabetes mellitus and obesity, poor self-rated health, heart disease and respiratory disease, to name a few. In their study, the highest associations were found for child maltreatment with sexual risk-taking, mental disorders, problematic alcohol use and interpersonal and self-directed violence (49). Furthermore, child maltreatment is negatively associated with adult education, employment, and income potential (50). Strengthening the understanding of the combined effects of child maltreatment and physical diseases may induce multidisciplinary prevention programs focused on prevention. If child maltreatment is a relevant factor in developing mental disorders and quality of life, a short questionnaire concerning early traumatization may be part of a multidisciplinary approach to the patient.

### Quality of Life

QoL is a complex phenomenon comprising different levels of physical, mental, and social levels of functioning (2) that has been the topic of many studies on ACHD. Two previous studies have shown that in ACHD patients, poor QoL is associated with the presence of mental disorders (7), older age, lack of employment, disability, no history of marriage, and poor NYHA class (1). However, both mentioned studies did not include any information on the presence of child maltreatment, which could influence the incidence and severity of mental disorders, thereby decreasing QoL. Hence, our study is the first that revealed a new factor that could influence the incidence of mental disorders and QoL in ACHD.

## Limitations

To the best of our knowledge, the present study is the first study that addressed child maltreatment as assessed with the CTQ in ACHD. However, our study has some limitations that need to be discussed. We cannot deduce a causal relationship between reported child maltreatment and mental health because of our data's correlative nature. Further prospective studies in children with CHD may overcome this methodological flaw. Also, the reported child maltreatment values could be influenced by a recall bias (17), a general problem in retrospective studies. Finally, no assessment was done on the prevalence of mental disorders in the patients' parents, and thus the possible relationship between the parents' mental disorders and child maltreatment in the present sample remains unclear.

## Conclusion

Child maltreatment, particularly sexual abuse, emotional neglect and emotional abuse, is more common in patients with ACHD. Total child maltreatment is associated with worse perceived quality of life and is associated with adverse physical (NYHA scores) and mental health (depression, anxiety and quality of life) outcomes, namely cardiac function and depression as well as anxiety and quality of life. Screening adult patients for history of child maltreatment can help both patients and professionals understand the underlying causes of health problems and enable better-informed treatment options. Psychosocial interventions that have proven efficacy in the context of trauma-related disorders (51, 52) can be applied to patients with a history of child maltreatment. A multidisciplinary approach to the patient that integrates past experiences and psychological problems is recommended in ACHD.

## Data Availability Statement

The raw data supporting the conclusions of this article will be made available by the authors upon request, without undue reservation.

## Ethics Statement

The studies involving human participants were reviewed and approved by Ethics Committee, Hannover Medical School, Hannover, Germany. The patients/participants provided their written informed consent to participate in this study.

## Author Contributions

PP and KK wrote the initial draft of the manuscript. MW-B and KK contributed to conception and design of the study. PP and IH performed the statistical analysis and revised the initial draft of the manuscript. All authors contributed to the final version and approved the manuscript.

## Conflict of Interest

The authors declare that the research was conducted in the absence of any commercial or financial relationships that could be construed as a potential conflict of interest.

## Publisher's Note

All claims expressed in this article are solely those of the authors and do not necessarily represent those of their affiliated organizations, or those of the publisher, the editors and the reviewers. Any product that may be evaluated in this article, or claim that may be made by its manufacturer, is not guaranteed or endorsed by the publisher.
